# Report of the Supervising Surgeon-General of the United States Marine-Hospital Service, 1887

**Published:** 1888-01

**Authors:** 


### EXTRACTS FROM THE FIFTEENTH ANNUAL REPORT OF THE SUPERVISING SURGEON-GENERAL OF THE UNITED STATES MARINE-HOSPITAL SERVICE, 1887.

### RELIEF FURNISHED.

| | |
|---|---|
| Patients relieved | 45,314 |
| Treated in hospitals | 13,084 |
| Treated in dispensaries | 32,230 |
| Days' relief in hospital furnished | 331,701 |

This is the largest number of patients furnished relief in any year since the organization of the service.

### THE QUARANTINE SERVICE.

*Vessels Boarded and Inspected at the Several United States Quarantine Stations.*

| | October, 1886, to April, 1887. | 1887. | | | | | |
|---|---|---|---|---|---|---|---|
| | | April. | May. | June. | July. | August. | Sept. |
| Delaware Breakwater | ........ | ........ | ........ | 45 | 49 | 22 | 29 |
| Cape Charles | ........ | 7 | 17 | 7 | 11 | 17 | 70 |
| South Atlantic (Sapelo Sound) | ........ | ........ | 1 | ........ | 1 | ........ | ........ |
| Ship Island (Gulf of Mexico) | 22 | 5 | 11 | 11 | 12 | 7 | 10 |

The station at Ship Island has been kept open the year round. The hospital at Delaware Breakwater is also kept open during the year, but the inspection of vessels is discontinued at the end of the quarantine season, usually October 31. The other stations are closed during the winter and placed in charge of keepers.

The stations at Delaware Breakwater and Cape Charles serve not only as protection to the several large cities and the States in the immediate vicinity, but through them to the country at large, and the recommendation made in previous reports that these stations be thoroughly equipped with all the modern appliances of quarantine, is respectfully renewed. Special appropriations are required to provide additional buildings and wharf facilities. Extensive repairs are also needed at Ship Island, or, in lieu thereof, a new plant on one of the other islands in the Gulf. One of the Chandeleurs, or the Grand Gozier, was recommended in my report for 1883, or the Pass à l'Outre, which would doubtless be the best location for the national quarantines. A bill for this purpose passed the Senate February 2, 1887, but, although reported back to the House favorably by the Committee on Commerce, it was not acted upon. The great importance of this measure leads me to hope that it will pass at the next session.

AID TO STATE AND LOCAL BOARDS OF HEALTH.

The several stations on the Atlantic and Gulf coasts should be properly equipped, and a station established on the Pacific coast, in San Francisco bay. Then the efficiency of the services contemplated by the act of April 29, 1878—aid to State and local boards—would be greatly increased.

The following letter has been received in regard to the Delaware Breakwater quarantine station :

STATE BOARD OF HEALTH,
EXECUTIVE OFFICE, 1532 PINE STREET,
PHILADELPHIA, *August* 3, 1887.

DEAR SIR : I am instructed by the State Board of Health of Pennsylvania to transmit to you the following resolution, passed at the stated meeting held July 13, 1887.

I have the honor to be,
Yours, very respectfully,
BENJ'N LEE, *Secretary.*

DR. JOHN B. HAMILTON,
*Supervising Surg.-Gen., U. S. M. H. S.*

*Resolved*, That, in the opinion of this board, the quarantine facilities now existing on the Delaware bay and river are insufficient in extent and antiquated in character, and this board, therefore, petitions the Government of the United States to complete the partial and inadequate arrangements which now exist at the mouth of Delaware bay, and to extend the term of quarantine against small-pox throughout the entire year.

WEEKLY ABSTRACT OF SANITARY REPORTS.

The publication of the weekly abstract of sanitary reports, as required by section 4 of the above-mentioned act, was resumed in January last and is being continued. Copies of each abstract are sent to the medical officers and acting assistant surgeons of the Marine-Hospital Service, customs officers, health officers of the various ports, State and local boards of health, and numerous sanitarians throughout the country.

In accordance with the provision contained in the sundry civil appropriation act, the method practiced in Mexico and Brazil for inoculation as a protection against yellow fever is now being investigated by Major George M. Sternberg, Surgeon U. S. Army, who has been detailed by the President of the United States for that investigation. He has been provided by this bureau with the necessary appliances, on his requisition, and it is believed that his report will be handed you in the course of the winter. Dr. Freire's own statement is now public as to the method claimed by him to have been successful in Brazil, and can be found in the Transactions of the International Medical Congress. The general opinion of sanitarians, however, has not yet crystallized in favor of inoculation as a preventive of yellow fever. The experiments made on the subject in Havana in 1855 were seemingly as conclusive as those of to-day, but they did not succeed in securing general adoption, and Dr. Sternberg's report will doubtless provide the necessary data for passing judgment on the efficacy of the methods.

CHOLERA IN EUROPE.

Cholera, although disappearing at one place only to reappear elsewhere, has lingered in Europe, principally in Italy, during the past year. The places from which the disease has been reported, in addition to those mentioned in the last annual report, are Cagliara, Rome, Castelamare, Genoa, St. Giovanni, Resina, Catania, Messina, Palermo, Valletta and Esseg, Austria-Hungary, and recent consular reports (August, 1887) show a rapid increase of the disease, especially in Rome, Tivoli, Valletta, Palermo, Messina, Resina, and Naples.

CHOLERA IN SOUTH AMERICA.

The disease made its appearance in Buenos Ayres in the month of November last, and was, as shown by the report of the United States minister, directly "imported from Genoa, Italy, by the Italian ship,

*Perseo*, plying between Genoa and Buenos Ayres. The envoy extraordinary and minister plenipotentiary of the Argentine Government in Italy was a passenger on the ship, and the anxiety to secure him an immediate landing on the part of the ship's commander, seems to have so far overcome his sense of duty that, by concealed or garbled reports, he managed to turn loose on Argentine soil, first here, then at Rosario, a great many persons from an infected ship. The testimony of passengers shows conclusively there was nearly a score of burials at sea of those who died of cholera on the voyage." During the month of November. notwithstanding the promptness in adopting sanitary measures, two hundred patients sick with cholera were admitted to the cholera hospital at Buenos Ayres, of which number ninety-three died and seventy-three were still under treatment at the end of the month.

In Rosario the disease was more fatal; in a population of 50,000 there were from thirty-five to fifty deaths per day. In the cholera hospital alone there were over two hundred patients in November, of which more than fifty per cent. proved fatal. A month later the reports from Buenos Ayres were more encouraging, but the disease had broken out in greater violence in the interior of the republic. In Mendosa, the development of the disease was most remarkable; the population of the city, 20,000, was almost decimated. At Tucuman, Cordova, Montevideo, Santiago, and Valparaiso, and especially along the valley of the Aconcagua following the course of the river towards the sea, the spread of the disease was exceptionally rapid. It was reported that cholera disappeared from Buenos Ayres in the month of March, 1887, but a number of cases was reported the succeeding month, and an official report dated as late as August 13, 1887, says that "cholera has never fully disappeared from the south of Chili, and that it is now increasing at Concepcion, and possibly at Talcahuano and Talca, and is causing much alarm on the coast."

### CHOLERA IN THE UNITED STATES.

The steamship *Alesia*, from Naples, Italy, arrived at the New York quarantine on the night of September 22, 1887, with four cases of cholera on board. Eight deaths from the same disease occurred on the voyage. Three cases were also imported on the steamship Britannia.

### NOTIFICATION FROM ABROAD.

The forthcoming regulations of the Department, I am informed, will contain full and complete instructions to United States consular officers in regard to prompt notification of the existence of epidemics in foreign countries, and of the departure of emigrants and vessels from infected ports. The aid in this matter, heretofore furnished by the Department of State, is very great, but it will be greater under the new regulations.

If Congress will only provide for the proper equipment and maintenance of the national quarantines the sanitary defenses against invasion of epidemic diseases will be greatly strengthened. The act of April 29, 1878, should be supplemented by new legislation providing a severe penalty for its violation. A vessel bringing cholera or yellow fever to our shores with the connivance of the officers, should not escape lightly.

### APPOINTMENTS.

One examining board was convened during the year for the examination of applicants for admission into the service, and of the seven candidates who passed the examination successfully, one was appointed an assistant surgeon, taking rank in the order of merit. The service is open to all regular graduates in medicine, between the ages of twenty-one and thirty years, without regard to political recommendations.

Acting assistant surgeons have been reappointed at Delaware Breakwater, Cape Charles and Gulf quarantines for duty during the quarantine season.

QUARANTINE HOSPITALS.

The hospitals at Ship Island, Sapelo Sound, and at Fisherman's Island are mere temporary structures, maintained from year to year from the contingent appropriation for the prevention of the spread of epidemic diseases. The hospital at the Delaware Breakwater is in better condition than either of the others, but it is out of the question to have first-rate hospitals on such an uncertain basis as that of contingent appropriations. If they are to be maintained at all, they should be well built and in such a manner as to insure the highest state of efficiency.

### REPORT OF SURGICAL OPERATIONS.

FISCAL YEAR 1887.

| Operations. | No. of cases. | Remarks. |
|---|---|---|
| **Total Number of Operations** | 613 | |
| REMOVAL OF TUMORS | 23 | |
| For sebacoaus cysts | 3 | Removed. |
| For cyst of neck | 2 | Aspiration and compression. |
| For keloid of jaw | 1 | Removed with knife. |
| For keloid of neck | 1 | Do. |
| For lipoma of back | 2 | Do. |
| For adenoma of neck | 1 | Do. |
| For epithelioma | 6 | Do. |
| For carcinoma, upper jaw | 1 | Removal of jaw; in hospital. |
| For sarcoma of orbit | 1 | Enucleation. |
| For lipoma of orbit | 1 | Removed with knife. |
| For melano-sarcoma of choroid | 1 | Enucleation. |
| For synovial cyst of wrist | 1 | Removed with knife. |
| For non-maligant growth of eyelid | 1 | Do. |
| For colloid carcinoma of breast | 1 | Do. |
| REMOVAL OF FOREIGN BODIES | 6 | |
| For gunshot wound of chest | 1 | Ball removed. |
| For gunshot wound of hand | 2 | Do. |
| For gunshot wound of thigh | 1 | Do. |
| For splinter in thigh | 1 | Splinter removed. |
| For needle in arm | 1 | Needle removed. |
| OPENING OF ABSCESSES | 10 | |
| For abscess of liver | 2 | Incision, 1; aspiration, 1; died, 1. |
| For abscess, scrotum and perineum | 1 | Died; pyæmia. |
| For abscess, perityphlitic | 1 | Incision and drainage. |
| For abscess, perinephritic | 1 | Do. |
| For abscess, psoas | 1 | Aspiration; recovery. |
| For abscess, axilla | 1 | Incision and drainage. |
| For abscess, chest wall | 1 | Incision and curetting. |
| For abscess, forearm, dissecting | 1 | Incision. |
| For abscess, ischio-rectal | 1 | Incision and drainage. |
| OPERATIONS ON THE NERVES | 3 | |
| For neuralgia, fifth nerve | 2 | Section of inf. maxillary nerve, 2.5 cm. removed, 1; neurotomy left sup. max. div., 1. |
| For neuritis, popliteal branches | 1 | Stretching popliteal nerve; successful. |
| OPERATIONS ON THE EYE AND APPENDAGES | 12 | |
| For entropion | 1 | |
| For pterygium | 5 | Excision. |
| For lachrymal abscess | 1 | Incision. |
| For prolapse of iris | 1 | Iridectomy. |
| For cataract | 1 | Flap operation. |
| For chalazion | 1 | Excision. |
| For ulcer of cornea | 1 | Iridectomy. |
| For leucoma | 1 | Do. |
| OPERATIONS ON THE HEAD | 2 | |
| For fracture of the skull, punctured | 1 | Trephined; recovered. |
| For Dermoid cyst, frontal bone | 1 | Extirpation. |
| OPERATIONS ON THE FACE AND MOUTH | 10 | |
| For removal of tonsils | 1 | Hypertrophy. |
| For elongation of uvula | 2 | Excision. |
| For necrosis, upper jaw | 4 | Dead bone removed. |
| For necrosis, lower jaw | 1 | Do. |
| For cleft palate | 1 | Unsuccessful. |
| For necrosis of turbinated bones | 1 | Crushed and removed. |
| OPERATIONS ON THE ARTERIES | 3 | |
| For aneurysm, popliteal artery | 2 | Compression, 1 failed; ligation, 1 successful. |
| For wound of temporal artery | 1 | Ligation. |

SURGICAL OPERATIONS, FISCAL YEAR 1887—*Continued.*

| Operations. | No. of cases. | Remarks. |
|---|---|---|
| OPERATIONS ON THE VEINS | 2 | |
| For varicose veins of leg | 2 | Successful, 1; unsuccessful, 1. |
| OPERATIONS ON THE RESPIRATORY ORGANS | 23 | |
| For hydrothorax | 14 | Aspiration; two died. |
| For empyema | 7 | Paracentesis and excision of rib; all recovered. |
| For abscess of lung | 1 | Aspiration; not successful. |
| For hæmatoma | 1 | Do. |
| For œdema of glottis | 1 | Laryngotomy; recovery. |
| OPERATIONS ON THE DIGESTIVE ORGANS | 66 | |
| For hernia, ingunial, strangulated | 3 | Two died. |
| For hernia, radical cure | 11 | One died. |
| For fistula in ano | 20 | Incision, 19; ligature, 1. |
| For hæmorrhoids | 26 | Ligature, 14; injection carbolic acid, 7; clamp and cautery, 3. |
| For ascites | 6 | Three died. |
| OPERATIONS ON THE LYMPHATIC ORGANS | 16 | |
| For removal of diseased lymph-glands | 16 | Dissected out. |
| OPERATIONS ON THE URINARY ORGANS | 156 | |
| For pyonephrosis | 2 | Nephrotomy; successful. |
| For retention of urine | 1 | Aspiration of bladder; died. |
| For vesical calculus | 3 | Lateral lithotomy, 1; median, 2; all recovered. |
| For stricture of the urethra | 147 | |
| *a.* Gradual dilatation | 14 | |
| *b.* Forcible dilatation | 52 | |
| *c.* Internal urethrotomy | 69 | 3 died: 2, pyæmia; 1, shock. |
| *d.* External urethrotomy | 5 | 1 died; pyæmia. |
| *f.* Perineal section | 1 | Recovered. |
| *g.* Slitting meatus | 3 | |
| For urinary fistula | 2 | Plastic, 1; not successful, ext. urethrotomy, 1. |
| For cystocele | 1 | Emmet's operation. |
| OPERATIONS ON THE GENERATIVE ORGANS | 113 | |
| For phymosis | 70 | 11 slitting; 59 circumcision. |
| For hydrocele | 28 | 20 radical cure. |
| For hydrocele of cord | 4 | Radical cure. |
| For sarcoma, testicle | 1 | Castration. |
| For orchitis, chronic | 1 | Do. |
| For syphilitic disease of testicle | 1 | Do. |
| For tubercular disease of testicle | 3 | Do. |
| For cystic disease of testicle | 2 | Do. |
| For hæmatocele | 1 | Incision. |
| For epithelioma of penis | 1 | Amputation. |
| For primary syphilitic disease of penis | 1 | Do. |
| OPERATIONS ON THE ORGANS OF LOCOMOTION | 63 | |
| ON BONES | 22 | |
| For caries of sternum | 3 | Dead bone removed. |
| For fracture, elbow joint | 1 | Excision olecranon. |
| For necrosis of ulna | 3 | Dead bone excised. |
| For caries of radius and ulna | 1 | Bones scraped. |
| For caries of pelvis | 2 | Scraping. |
| For necrosis of humerus | 1 | Removal necrosed bone. |
| For necrosis of patella | 1 | Do. |
| For necrosis of tibia | 4 | Do. |
| For necrosis of femur | 1 | Trephine and gouge. |
| For vicious union, bones of forearm | 1 | Refracture. |
| For ununited fracture of tibia | 3 | 2 unsuccessful. |
| For faulty union, fractured femur | 1 | Refracture. |
| ON JOINTS | 41 | |
| Inflammation of hip | 1 | Resection. |
| Reduction of dislocations: | | |
| Jaw | 2 | Manipulation. |
| Shoulder | 2 | Do. |
| Clavicle, sternal | 1 | Do. |
| Elbow | 2 | Do. |
| Hip | 1 | Do. |
| Ankylosis, elbow | 1 | Rupture. |
| Loose cartileges, knee-joint | 2 | Incision and removal. |
| Chronic synovitis, knee-joint | 24 | Aspiration. |
| Bursal inflammation | 3 | Do. |
| Bursal tumor | 1 | Removed. |
| Contraction extensor tendons of toes | 1 | Tenotomy. |
| AMPUTATIONS | 96 | |
| Fingers for contracted tendon | 1 | |
| Fingers for whitlow | 6 | |
| Fingers for frostbite | 3 | |
| Fingers for necrosis | 10 | |
| Fingers for abscess | 2 | |
| Fingers for caries | 4 | |
| Fingers for compound fracture | 7 | |
| Fingers for lacerated wound | 10 | |
| Fingers for burns | 1 | |
| Hand for compound com. fracture | 1 | |

SURGICAL OPERATIONS, FISCAL YEAR 1887—*Continued.*

| Operations. | No. of cases. | Remarks. |
|---|---|---|
| AMPUTATIONS—Continued. | | |
| Hand for osteitis | 1 | |
| Forearm for compound com. fracture | 1 | |
| Forearm for necrosis | 1 | |
| Forearm for caries, radius, and ulna | 1 | |
| Arm for compound fracture | 8 | 2 recovered; 1 died. |
| Toes for compound fracture | 2 | |
| Toes for frostbite | 21 | |
| Toes for gangrene | 1 | |
| Toes for necrosis | 4 | |
| Toes for periostitis | 1 | |
| Toes for wound | 1 | |
| Toes for ankylosis | 1 | |
| Foot for compound fracture | 1 | |
| Foot for frostbite | 4 | |
| Foot for lacerated wound | 1 | |
| Foot for necrosis | 1 | |
| Leg for compound com. fracture | 1 | Died; septicæmia. |
| Leg for necrosis | 2 | Both recovered. |
| Thigh for necrosis | 2 | Do. |
| OPERATIONS ON THE SKIN | 9 | |
| Chronic ulcer | 4 | Skin grafting. |
| Ingrown nail | 1 | Avulsion. |
| Onychia | 2 | Nail removed. |
| Contracted palmar fascia | 1 | Subcutaneous section. |
| Lac. wound of hand | 1 | Skin grafting. |

